# Characterizing the Milk Microbiome in Subclinical Mastitis: A Pilot 16S rRNA‐Based Study in Cattle and Water Buffalo

**DOI:** 10.1002/vms3.71154

**Published:** 2026-08-05

**Authors:** Md. Habib Ullah Masum, Maksudur Rahman Nayem, Ahmad Abdullah Mahdeen, Sumaia Sultana, Abanti Barua

**Affiliations:** ^1^ Department of Genomics and Bioinformatics Faculty of Biotechnology and Genetic Engineering Chattogram Veterinary and Animal Sciences University Khulshi Bangladesh; ^2^ NGS Division DNA Solution Ltd., Bridge Momtaz Heights Dhaka Bangladesh; ^3^ Department of Microbiology Notre Dame University Dhaka Bangladesh; ^4^ Department of Microbiology Noakhali Science and Technology University Noakhali Bangladesh

**Keywords:** amplicon sequencing, milk microbiome, Noakhali, Bangladesh, subclinical mastitis

## Abstract

**Background:**

In the dairy sector of Bangladesh, subclinical mastitis (SCM) is a substantial and frequently undiagnosed challenge, with reported prevalence rates of 60%–77% in cattle and approximately 52% in buffaloes. Due to its complex characteristics and progressive development, efficient diagnosis and management are essential for enhancing dairy productivity.

**Objectives:**

This pilot study employed 16S rRNA amplicon sequencing using Oxford Nanopore's MinION to investigate the milk microbiota of healthy and mastitic cattle and buffalo.

**Results:**

A total of 423 clustered nucleotide sequences were identified in the samples, indicating significant taxonomic diversity: 11 phyla, 26 classes, 58 orders, 120 families and 272 genera. Distinct phylum‐level patterns were observed, with Firmicutes predominating in healthy milk and a relative increase in Proteobacteria and Actinobacteriota in mastitic samples. At the genus level, *Streptococcus* and *Lactococcus* were predominant in mastitic samples, whereas *Staphylococcus* and *Lactococcus* were more prevalent in healthy milk. The results indicate that although overall microbial diversity was relatively consistent across groups, mastitis correlated with alterations in bacterial community composition, with notable differences between cattle and buffalo.

**Conclusion:**

This study suggests a potential association between SCM and microbial shifts; however, microbiome profiling cannot yet be recommended for diagnostic application. Clinical applicability requires validation in large‐scale studies with individual‐level sampling.

## Graphical abstract

Milk samples collected from cattle and buffalo were pooled and screened for mastitis prior to DNA extraction. Sequencing of the V1–V7 region of 16S rRNA was performed using the MinION platform, and the resulting data were analysed to determine taxonomic classification and assess microbial diversity.

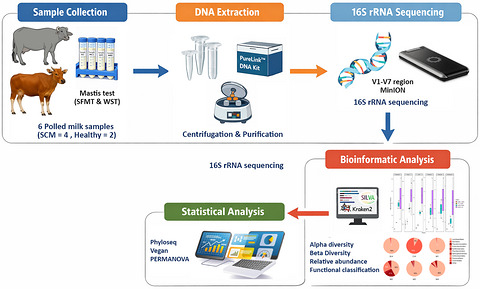

## 1 Introduction

Mastitis, the inflammation of the mammary gland, is one of the most prevalent diseases affecting sheep and other dairy animals (De Vliegher et al. 2012; Ali et al. 2021). The disease is the highly detrimental to the dairy sector, resulting in substantial financial losses, undermining animal welfare and health, and adversely affecting milk quality and quantity (Ali et al. 2021). This condition has been associated with substantial variations in milk chemistry, including a significant decrease in milk output and alterations to cell permeability (Ali et al. 2021; Schukken et al. 2009). The estimated overall cost of bovine mastitis is approximately $147 per cow per year, primarily due to reduced milk yield and culling, which accounts for 11%–18% of the annual gross margin per cow (Hogeveen et al. 2019; Cheng and Han 2020). On the other hand, the average annual economic loss per buffalo attributable to mastitis was $70, with mastitis interventions accounting for 55% of the cost and a decrease in milk output accounting for 16% (Singha et al. 2023; Malik and Verma 2017). Bangladesh has roughly 6 million dairy cattle, mostly crossbred high‐yielding cattle, producing over 9.4 million metric tons of milk annually (Emon et al. 2024). In this region, approximately 70% of farmers are smallholders, each managing one to three cattle per farm. Despite their small‐scale operations, these farmers collectively contribute 70%–80% of the country's total milk production (Uddin et al. 2012). However, the productivity of domestic dairy cattle remains relatively low, with an average milk yield of only 200–250 L per 305‐day lactation period (Kong et al. 2018). Production illnesses, including mastitis, are the most significant barrier to a dairy farm's capacity for optimal profitability (Zaatout and Hezil 2022). In Bangladesh, mastitis is estimated to cause an annual loss of Tk122.6 million ($2.11 million) (Kabir et al. 2019).

Mastitis infections may be categorized as clinical mastitis (CM), subclinical mastitis (SCM) and recurrent mastitis (RCM) depending on the severity of the illness (Abd El‐Razik et al. 2023). CM is evident and often recognized in dairy animals by physical indicators such as erythema and oedema of the udders, accompanied by pyrexia. In addition, floaters and clots are discernible in mammalian milk, which has a watery consistency (Khan and Khan 2006). However, this condition can be fatal in extreme circumstances and is categorized as per‐acute, acute and sub‐acute according to the intensity of the inflammation (Gruet et al. 2001). CM significantly impacts the financial health of dairy farms due to issues such as abnormal milk, reduced milk quality, a drop in production (up to 70%), milk disposal post‐treatment (9%), treatment costs (7%), labour expenses, early culling (14%) and mortality rates (Cheng and Han 2020; Kabir et al. 2019; Abd El‐Razik et al. 2023). On the other hand, SCM is characterized by the lack of clinical symptoms, unlike the rise in the somatic cell count of the milk. Research shows that SCM is significantly more prevalent, with estimates of 15–40 times that of CM (Emon et al. 2024). Owing to its often obscure diagnosis, experts contend that it may result in greater economic losses within livestock herds than overt CM (Romero et al. 2018). Mastitis is mostly (70%) caused by bacterial infections. Similarly, non‐infectious factors like physical trauma and mechanical injury to the gland account for 30% of the minor contributors (Bradley 2002). More than 135 distinct bacterial species have been implicated in bovine mastitis, although the most frequent pathogenic bacteria in dairy cattle mastitis are 20 distinct species (Bradley 2002; Gao et al. 2017). *Staphylococcus aureus*, *Escherichia coli*, coagulase‐negative staphylococci (CNS), *Streptococcus dysgalactiae, Streptococcus uberis* and *Streptococcus agalactiae* are the most prominent mastitis‐causing bacteria (Gao et al. 2017; Ali et al. 2016; Yang et al. 2020; Ali et al. 2021; Tadee et al. 2025).

Correctly identifying the bacterial pathogens responsible for mastitis is crucial for selecting appropriate and effective antimicrobial treatments, particularly given the growing problem of antibiotic resistance and the varying susceptibility of pathogens. Antibiotics such as streptomycin, ampicillin, cloxacillin and tetracycline have traditionally served as primary treatments (Pascu et al. 2022; Bhosale et al. 2014), but misuse and overuse of antimicrobials have caused herd‐ or pathogen‐specific resistance (Kumari et al. 2018; Royster and Wagner 2015). Without proper identification, empirical treatments may fail, increasing the likelihood of resistance development and treatment failure. The evolution of multidrug‐resistant strains poses substantial public health risks, especially through the ingestion of unpasteurized milk contaminated with resistant microorganisms (Beyene et al. 2017; Abrahmsén et al. 2014; Oliveira and Ruegg 2014). Consequently, rapid and precise identification of the bacteria responsible for mastitis is essential to enhance therapeutic outcomes, mitigate economic losses and protect public health by preventing the spread of antimicrobial resistance (Oliveira and Ruegg 2014; Stevens et al. 2016; Ruegg 2009). The microbiological culture approach is regarded as the gold standard for pathogen identification in mastitis, yet bacterial DNA can be detected in culture‐negative samples from mastitic animals (Catozzi et al. 2020; Kuehn et al. 2013). Alterations in milk microbiota during mastitis or lactation stages have been reported in cattle (Lima et al. 2018; Metzger et al. 2018), goats (McInnis et al. 2015) and water buffalo (Catozzi et al. 2017). Conventional short‐read sequencing platforms, such as Illumina, typically target only one or a limited number of hypervariable regions within the 16S rRNA gene, which may constrain taxonomic resolution and hinder the accurate discrimination of closely related microbial taxa (Callahan et al. 2019). Due to the restricted taxonomic resolution of sequencing a short fragment of the 16S rRNA gene, full‐length 16S rRNA gene sequencing using single‐molecule sequencers may provide an approach for achieving species‐level identification and enhancing the characterization of milk microbiota from healthy and mastitis‐affected quarters. In contrast, third‐generation sequencing technologies, including Oxford Nanopore Technologies and Pacific Biosciences, are capable of generating near‐full‐length or full‐length 16S rRNA gene sequences (Callahan et al. 2019). This expanded sequence coverage provides greater phylogenetic information, thereby enhancing taxonomic classification and improving the characterization of complex microbial communities (Catozzi et al. 2020; Catozzi et al. 2017).

This pilot study aims to characterize the milk microbiota in cattle and water buffalo with SCM. To characterize the bacterial composition of pooled milk samples, we used full‐length 16S rRNA sequencing with Oxford Nanopore's MinION. This approach enables species‐level identification of milk microbiota and provides preliminary insights into microbial shifts associated with mastitis and lactation, which may inform future studies on milk quality and management.

## 2 Methods

### 2.1 Sample Collection

A total of six pooled milk samples were collected from local dairy farms in the Noakhali district, Bangladesh (M_C = 2 pools; M_B = 2 pools; C_H = 1 pool; B_H = 1 pool). Each pool contained 10 individual samples from a distinct farm. Samples were pooled according to farm and health‐status group to generate representative herd‐level microbial profiles and minimize inter‐individual variability, consistent with the exploratory pilot design of the study. Before sampling, the mastitis infection was confirmed by the district veterinary surgeon and then verified using two rapid mastitis tests: Surf Field Mastitis Test (SFMT) (Muhammad et al. 2010) and Wide Side Test (WST) (Bhat et al. 2014; Islam et al. 2011). The milk samples were sent to the Department of Microbiology at Noakhali Science and Technology University (NSTU) for additional experimentation at 4°C, and they were subsequently stored at −20°C.

### 2.2 DNA Extraction and Purification

The genomic DNA from the mastitic milk samples of both diseased and healthy hosts was extracted for six representative pooled samples (M_C = 2, M_B = 2, C_H = 1, and B_H = 1) using the commercially available PureLink Microbiome DNA Purification Kit (Cat no. A29790) following the manufacturer's instruction. The PureLink Microbiome DNA Purification Kit, although primarily marketed for saliva and urine samples, is optimized for low‐biomass and inhibitor‐rich biological fluids. Milk constitutes a complex matrix with fats, proteins, calcium and PCR inhibitors that can impede DNA extraction. Previous microbiome studies have demonstrated the successful application of similar extraction chemistries to milk and dairy samples for 16S rRNA gene‐based microbial profiling (Endres et al. 2021; Sanjulián et al. 2021). Prior to DNA extraction, milk samples were centrifuged at 10,000 × *g* for 10 min, yielding a top fat layer, a middle supernatant and a cellular pellet. The upper fat layer was removed, and the cell pellet was washed with 1× phosphate‐buffered saline (PBS) to facilitate recovery of purified DNA. The purified DNA samples were then stored at −80°C until further use.

### 2.3 16S rRNA Gene Sequencing on the MinION Platform

The V1–V7 region of the 16s rRNA gene was amplified using 27F and 1100R primer pair (Klindworth et al. 2013). PCR amplification of 16S rRNA genes was performed using Q5 High‐Fidelity 2X Master Mix according to the manufacturer's instructions. Amplification was performed with the following PCR conditions: initial denaturation at 95°C for 3 min, 30 cycles of 95°C for 15 s, 60°C for 15 s and 72°C for 60 s, followed by a final extension at 72°C for 10 min. Amplified DNA was purified using AMPure XP reagent (Beckman Coulter, Inc.) and quantified by Qubit 4 using Qubit 1X dsDNA High Sensitivity (HS) reagent (Thermo Fisher Scientific, USA). A total of 200 ng of DNA was utilized for end preparation and dA tailing, followed by the ligation of barcodes using the Native Barcoding Kit 24 V14 (SQK‐NBD114.24) from Oxford Nanopore. Using the NEBNext Quick Ligation Module (NEB, E6056), the sequencing adapter was ligated, and the prepared DNA libraries were aggregated. The complete adapter‐ligated and purified library, coupled with 37.5 µL of Sequencing Buffer and 25.5 µL of Loading Beads, was introduced into the R10.4.1 flow cell (FLO‐MIN106; Oxford Nanopore Technologies) and sequenced with the MinION Mk1C. Data collection was conducted with MinKNOW software version 1.11.5 from Oxford Nanopore Technologies.

A positive control and a negative control were processed alongside the study samples to evaluate taxonomic assignment accuracy and potential reagent‐ or laboratory‐derived contamination. The positive control consisted of DNA product from a culture‐confirmed bacterial sample and was included to assess concordance between culture‐based identification and Nanopore 16S‐based taxonomic classification. The negative control contained nuclease‐free water template and was processed through the same extraction, amplification, library preparation, sequencing and bioinformatic workflow as the milk samples. Control reads were subjected to the same basecalling, barcode/adaptor trimming, quality filtering, length filtering and taxonomic classification parameters used for the study samples. Taxa were considered potential contaminants if they were detected in the negative control and their abundance in biological samples was ≤ 10‐fold higher than in the negative control, or if they represented low‐abundance taxa near the minimum read threshold (10 reads). The positive control was used only for quality‐control validation and the negative control was used only for contamination assessment; neither control was included in alpha‐diversity, beta‐diversity, relative abundance or differential composition analyses.

### 2.4 Bioinformatic Analysis

Guppy (v6.3.2) with high accuracy mode was applied for basecalling the MinION Mk1C sequencing data (FAST5 files) to generate pass reads (FASTQ format) with a mean quality score > 9. Raw sequencing reads were processed using Porechop (v0.2.4) for adapter and barcode trimming (Wick and Volkening 2018). A barcode threshold score of 80 and an adapter threshold score of 95 were applied to maximize trimming accuracy. Following adapter trimming, sequencing reads were subjected to quality and length filtering using NanoFilt (v2.8.0) (De Coster et al. 2018). Reads with lengths between 800 and 1200 bp and a minimum mean quality score of 10 were retained for downstream analyses. This size range was selected to correspond to the expected amplicon length of the 16S rRNA V1–V7 region. Taxonomic assignment of the reads was performed using the wf‐16s workflow in EPI2me software (Oxford Nanopore Technologies) (Tyler et al. 2018) using SILVA 138.1 (Quast et al. 2013) as the reference database and Kraken2 (v.2.1.3) as the classifier (Wood et al. 2019). All other parameters were retained at their default settings. Taxonomic classification was performed with a confidence threshold of 0.1 (Kraken2), followed by abundance estimation with a minimum threshold of 10 reads (Kraken). Taxonomic assignments were subsequently summarized at the genus level for downstream microbial community analyses.

### 2.5 Statistical Analysis

The Phyloseq (v 4.2) (McMurdie and Holmes 2013), Vegan (Oksanen et al. 2015), ggplot2, ggpubr and microbiomeutilities packages for R (v 4.3.0) were used for downstream analysis, including alpha‐ and beta‐diversity, microbial composition, differential abundance calculation and statistical comparison. The clustered nucleotide sequence data were normalized by the total sum scaling technique (TSS), and the normalized data were used to measure alpha and beta diversity. Rarefaction curve from filtered taxonomic data is attached in Figure S1. The MetaGenAssist utility was employed to predict functional annotations (Arndt et al. 2012).

## 3 Results

### 3.1 Sequencing Control Performance and Contamination Assessment

Sequencing control analysis was performed to evaluate potential false‐positive taxonomic assignments and background contamination. The positive control showed strong agreement with the expected culture‐based identification, with 99.39% of classified reads assigned to the expected bacterial taxon. The negative control generated only a single read after quality and length filtering compared with the milk samples. The negative and positive controls were not included in diversity or compositional analyses and were used only for quality‐control assessment.

### 3.2 Bacteriome Diversity and Composition

To gain insight into the bacteriome signature and diversity in milk samples from cattle and buffalo across four groups—cattle mastitis (M_C), buffalo mastitis (M_B), healthy cattle (C_H) and healthy buffalo (B_H)— six pool milk samples were analysed using 16S rRNA amplicon sequencing. Table S1 includes the metadata of the study's sampling. In this study, a total of 423 clustered nucleotide sequences were identified across the sample groups.

**FIGURE 1:**
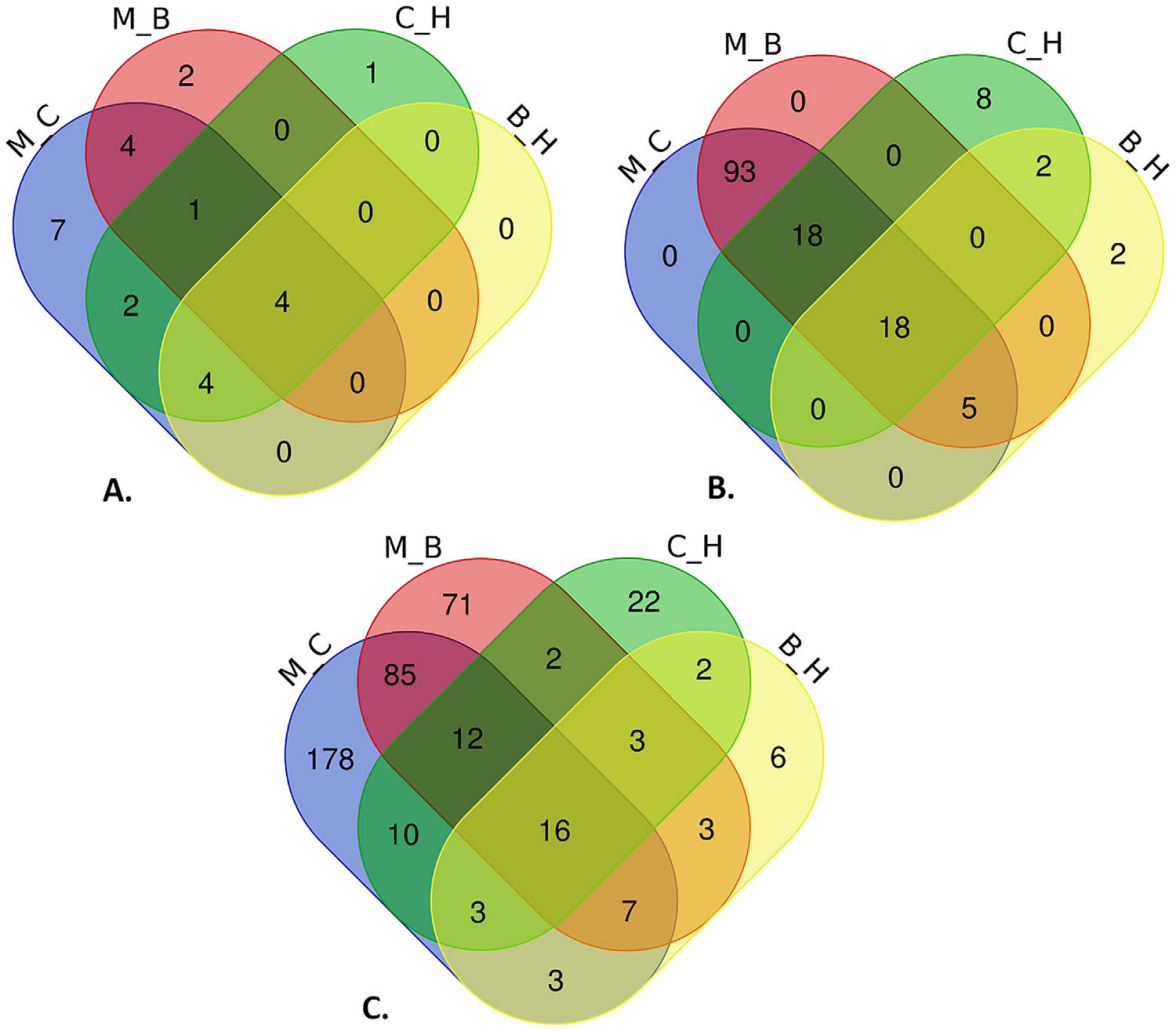
The composition of bacteriomes in terms of taxonomy. Venn diagrams representing the unique and shared bacterial (A) phylum, (B) family and (C) genus distributions identified in the sample groups.

The identified clustered nucleotide sequences included 11 phyla, 26 classes, 58 orders, 120 families and 272 genera of bacteria (Table S2). The unique and shared distribution of bacterial taxa found in the four sample groups (M_C, M_B, C_H and B_H) is represented in Venn diagrams (Figure 1). A total of 11 bacterial phyla were identified, of which 36.36% (4/11) were found to be shared among the M_C, M_B, C_H and B_H samples (Figure 1a, Table S2). However, seven particular phyla were found to be unique to the M_C group, whereas the M_B group had two unique phyla (Figure 1a, Data S1).

Also, a total of 120 bacterial families were identified, of which 15.0% (18/120) were found to be shared between M_C, M_B, C_H and B_H (Figure 1a). However, no particular family was found to be unique to the M_C and M_B groups (Figure 1a, Data S1). In terms of genera, a total of 272 bacterial genera were detected, of which 5.88% (16/272) were found to be shared between M_C, M_B, C_H and B_H (Figure 1a). However, 178 particular genera were found to be unique to the M_C group, whereas the M_B group had 71 unique genera (Figure 1a, Data S1, Table S2).

### 3.3 Relative Abundances of Bacteriomes

**FIGURE 2:**
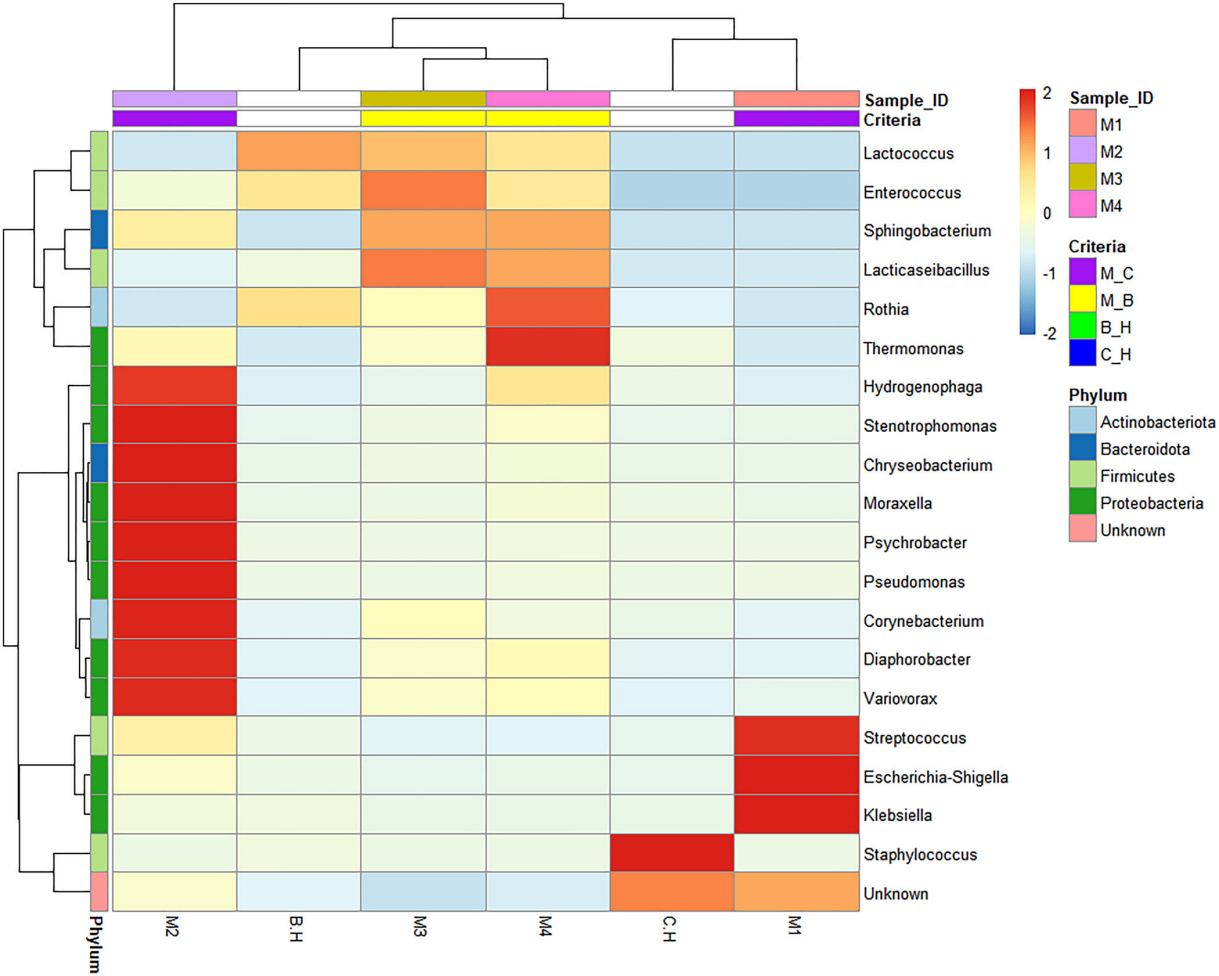
The relative abundances of bacteriome at phylum level. A heatmap is presented depicting the average relative abundances and hierarchical clustering of bacterial phyla (*n* = 20) throughout the study samples. The colour gradient from −2 to 2 signifies the abundance scale, where −2 represents the lowest abundance and 2 indicates the maximum. Red hues indicate bacterial phyla with greater abundance, whilst green hues signify phyla found in lesser proportions within the corresponding samples.

At the phylum level, Firmicutes (58.55%), Proteobacteria (15.09%) and Bacteroidota (12.00%) emerged as the predominant bacterial phyla in the M_C samples, collectively accounting for more than 85.64% of the total abundance (Figure 2, Data S1). Similarly, Firmicutes (73.58%), Actinobacteriota (19.55%) and Bacteroidota (3.40%) dominated the M_B samples, accounting for over 95.53% of the total bacterial abundance. When bacterial composition was analysed across sample categories, distinct patterns emerged. The C_H samples were primarily dominated by Firmicutes, which accounted for 79.48% of the total abundance. Notably, Firmicutes was even more prevalent in the B_H samples, accounting for a striking 97.31% of the bacterial community. In addition to the dominant phyla, other bacterial phyla with relative abundances of less than 2% showed significant variation across the sample categories, highlighting diverse microbial dynamics.

**FIGURE 3:**
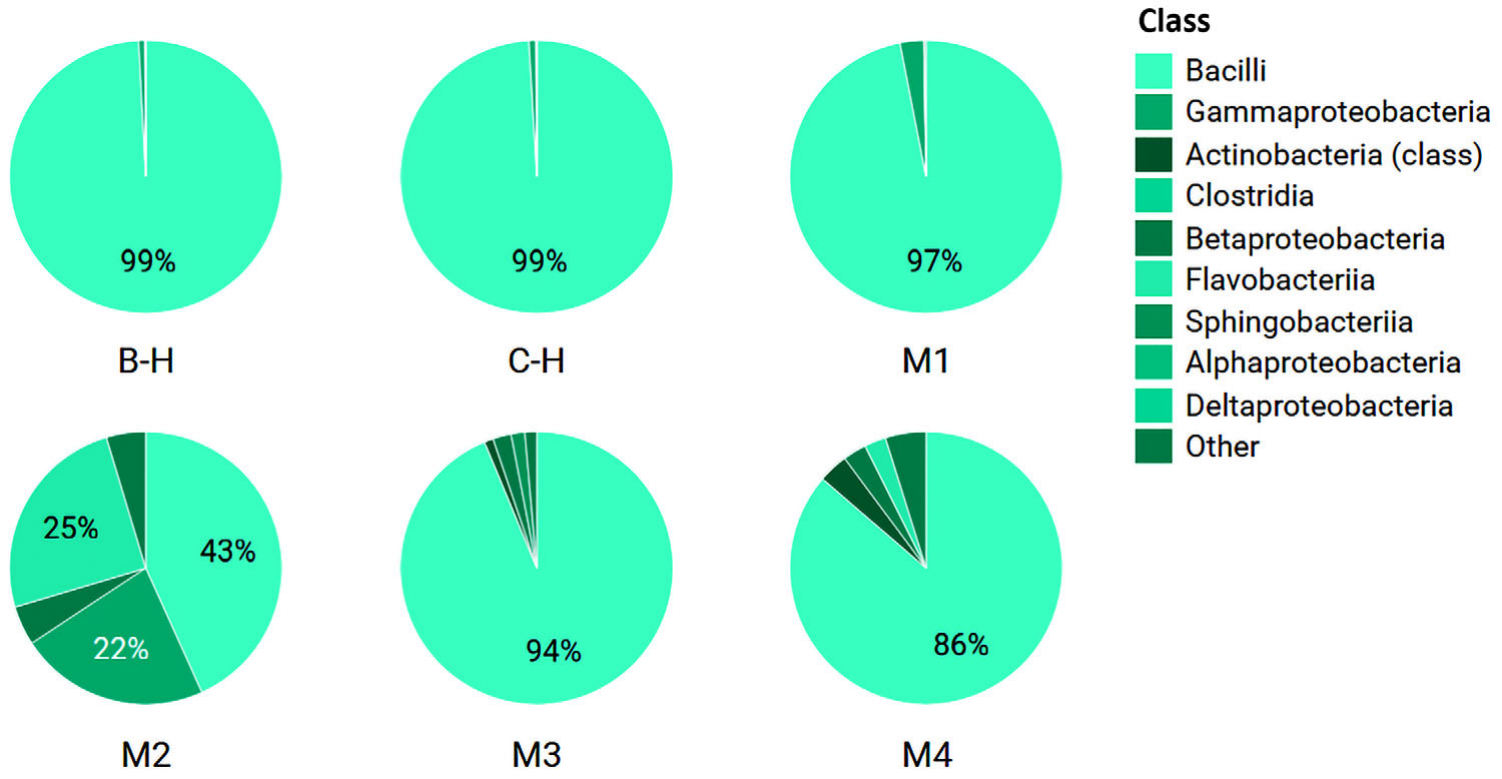
The relative abundances of bacteriome at the class level. The pie chart illustrates the relative abundance of the nine most prevalent bacterial classes within the sample groups.

In the M_C samples, Bacilli emerged as the most predominant bacterial class, accounting for 62.69% of the total community composition. This was followed by Flavobacteriia (15.91%), Gammaproteobacteria (15.36%) and Betaproteobacteria (2.97%). In contrast, the M_B samples showed a striking dominance of Bacilli, accounting for 90.58% of the bacterial community. In both the C_H and B_H samples, Bacilli emerged as the most predominant bacterial class, accounting for 99.02% and 99.14% of the total community composition, respectively. Beyond the dominant classes, bacterial classes with relative abundances below 2% showed notable variation across sample categories, underscoring the diverse microbial dynamics among the samples (Figure 3, Data S1).

**FIGURE 4:**
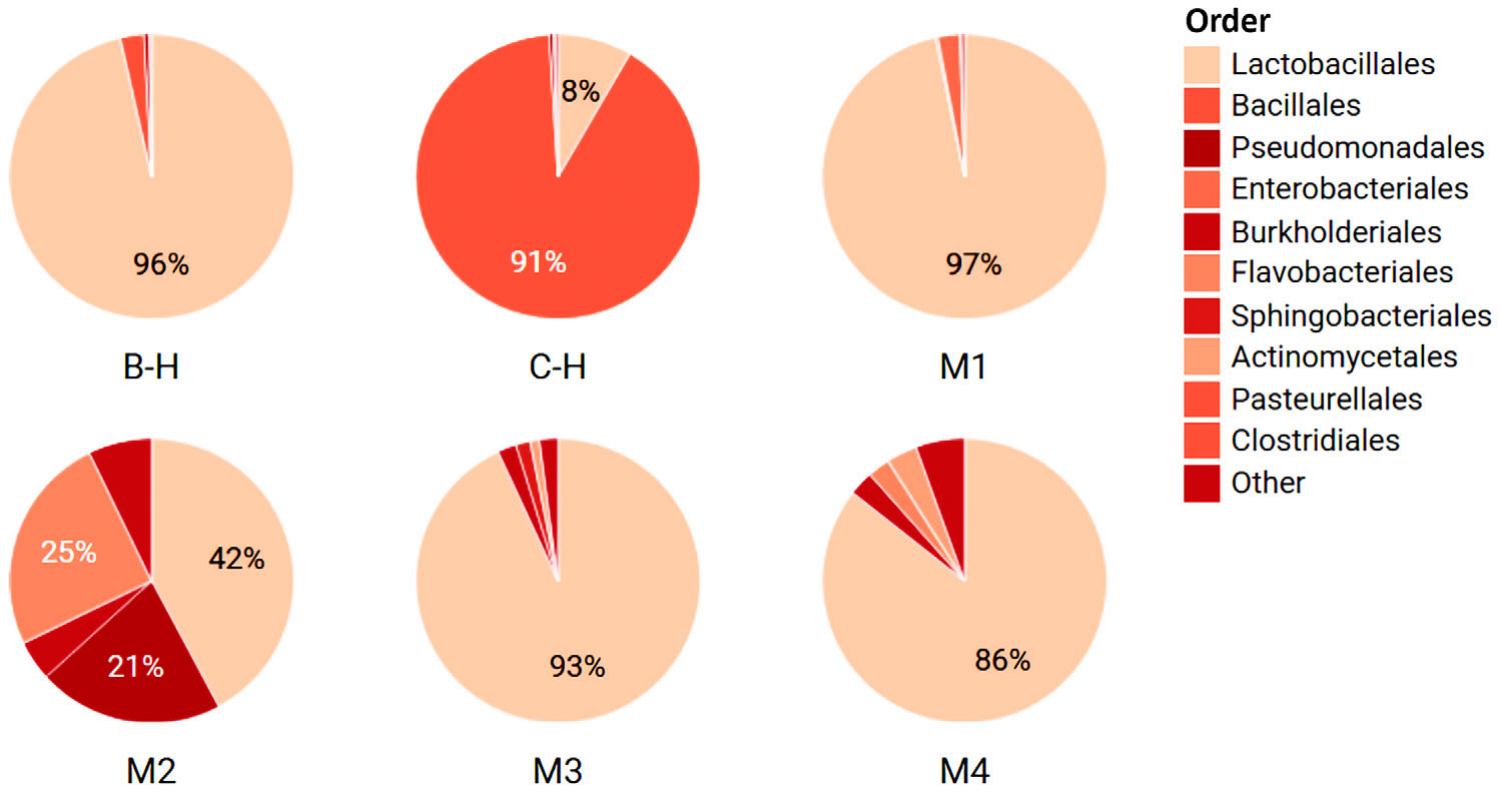
The relative abundances of bacteriome at the order level. The pie chart representing the relative abundance of the top 10 most abundant bacterial orders in the sample groups.

Among the predicted bacterial orders, Lactobacillales (61.98%), Flavobacteriales (15.93%), Pseudomonadales (13.44%) and Burkholderiales (2.90%) were identified as the most abundant in the M_C sample. In contrast, the M_B sample was dominated by Lactobacillales (89.93%), followed by Burkholderiales (2.40%) and Actinomycetales (2.12%). In the C_H samples, Bacillales emerged as the predominant bacterial order, accounting for 90.59% of the total composition, with Lactobacillales comprising 8.41%. Conversely, in the B_H samples, Lactobacillales was the most abundant order, followed by Bacillales (2.71%). Bacterial classes with relative abundances below 2%, aside from the dominant ones, displayed significant variation across the sample categories, highlighting the diverse microbial dynamics within the samples (Figure 4, Data S1).

**FIGURE 5:**
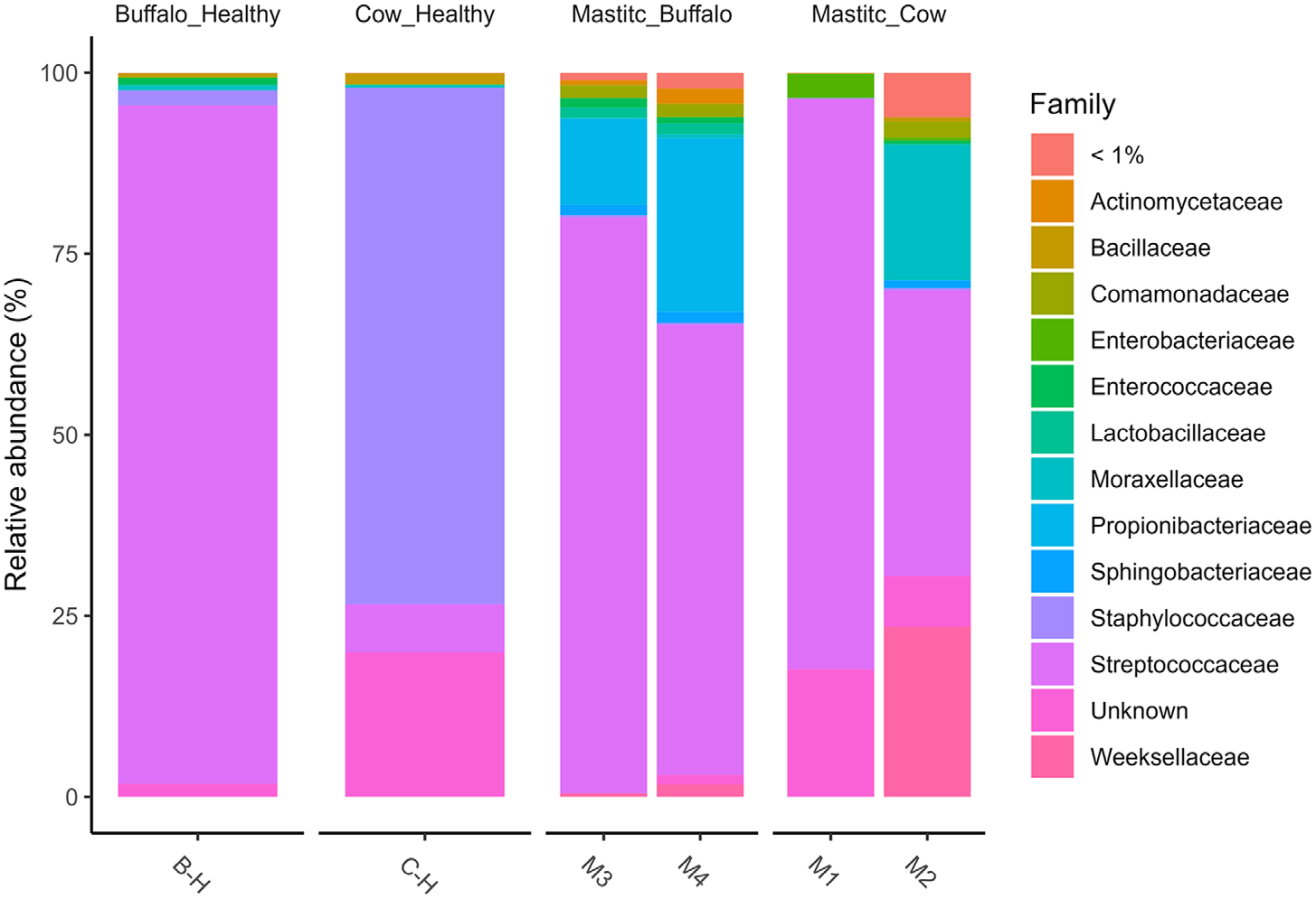
The relative abundances of bacteriome at the family level. The bar plots illustrate the relative abundance of the top 14 most prevalent bacterial families in the sample groups.

The family‐level composition and relative abundances of the bacteriome exhibited notable variations across the M_C, M_B, C_H and B_H sample types. In the M_C samples, Streptococcaceae emerged as the most abundant bacterial family, accounting for 57.49% of the total composition. This was followed by Weeksellaceae (11.05%) and Moraxellaceae (8.92%), highlighting a diverse bacterial community within this sample type. Similarly, in the M_B samples, Streptococcaceae showed staggering dominance, with a relative abundance of 88.47%. The next most prevalent family was Comamonadaceae, although it contributed only 2.09% to the total bacteriome (Figure 5, Data S1). Contrasting patterns were observed in the C_H samples, in which Staphylococcaceae was the most abundant family, accounting for 70.87% of the bacteriome. Streptococcaceae ranked second, with a relative abundance of 6.65%. The B_H samples were markedly dominated by Streptococcaceae, which accounted for 93.48% of the bacteriome composition. Staphylococcaceae was the second‐most prevalent family in this group, accounting for 2.08% of the total abundance. Other bacterial families across all sample types demonstrated relatively low abundances, with individual contributions averaging less than 2% (Data S1).

**FIGURE 6:**
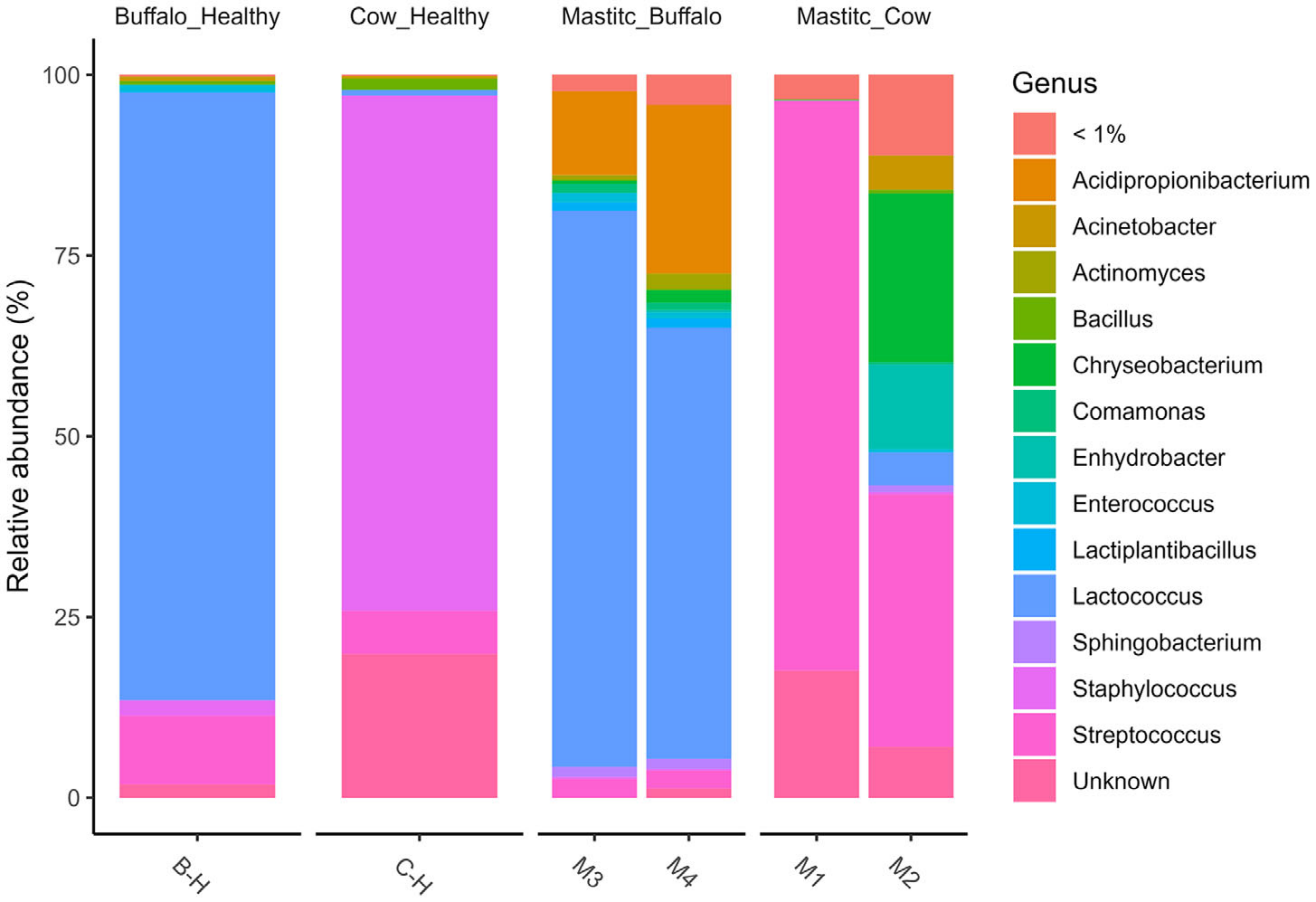
The relative abundances of bacteriome at genus level. The bar plots demonstrate the relative abundance of the 14 most abundant bacterial genera in the sample groups.

The genus‐level bacteriome composition and relative abundances showed significant differences across the M_C, M_B, C_H and B_H sample types. In the M_C samples, *Streptococcus* was the most dominant genus, representing 55.36% of the total composition. This was followed by *Chryseobacterium* (10.94%) and *Enhydrobacter* (5.47%), indicating a diverse bacterial community within this group. Conversely, the M_B samples were heavily dominated by *Lactococcus*, which accounted for 67.82% of the bacteriome. The second most abundant genus was *Acidipropionibacterium*, contributing 17.35% of the total composition (Figure 6, Data S1). A distinct pattern was observed in the C_H samples, in which *Staphylococcus* emerged as the predominant genus, accounting for 70.86% of the bacteriome, followed by *Streptococcus* at a much lower relative abundance of 5.90%. In the B_H samples, *Lactococcus* was the most abundant genus, accounting for 83.94% of the composition, with *Streptococcus* as the second‐most prevalent genus, contributing 9.54%. Across all sample types, other bacterial genera exhibited relatively low abundances, each accounting for less than 3% of the total composition (Data S1).

### 3.4 Functional Profiling

**FIGURE 7:**
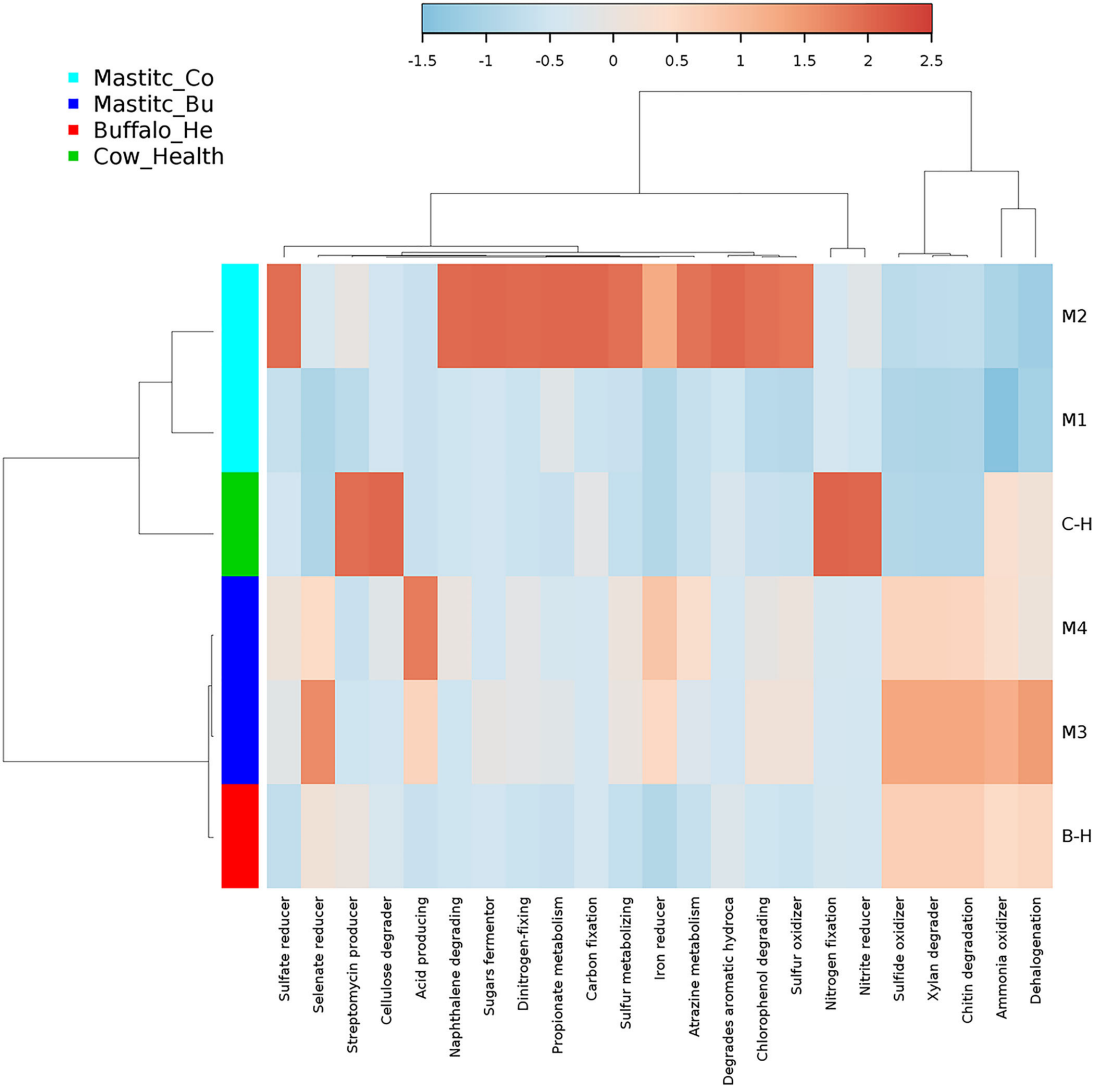
Bacteriome classified by metabolic functions. The heatmap shows the relative abundance of the classified bacteriomes by metabolic functions in the sample groups.

Functional profiling suggested variation in the relative abundance of predicted bacterial groups across sample categories. In the M_C group, dehalogenizer‐associated taxa represented 50.56% of the predicted functional profile, with ammonia oxidizers at 10.14%. The M_B group exhibited a comparatively balanced distribution of predicted functional groups, including ammonia oxidizers (19.96%), sulphide oxidizers (19.93%) and dehalogenizers (19.88%) (Figure 7, Data S2). The control groups also showed variation in predicted functional composition. Dehalogenizer‐associated taxa accounted for 25.67% of the C_H group and 21.46% of the B_H group, representing the largest proportion in these samples. Although differences in predicted functions were observed, these primarily reflect descriptive trends and require further validation.

## 4 Discussion

In Bangladesh, smallholder farmers account for the majority of the nation's milk production; however, productivity remains low due to factors such as mastitis. SCM is a particular concern due to its high prevalence and asymptomatic nature, which complicate detection and management (Datta et al. 2019). The worldwide prevalence of SCM in both cattle and buffaloes was 42% and 46%, respectively (Krishnamoorthy et al. 2021). In Bangladesh, the prevalence of SCM is reported to be around 60%–77% in cattle (Singha et al. 2021) and 52% in buffalo (Singha et al. 2023). The increased incidence of SCM considerably affects dairy production, animal production and farm output. This condition is influenced by multiple variables, including hygiene, lactation stage, parity, milking methods and heredity, with a global range of 20%–80% and an average of 50% (Bari et al. 2022). Therefore, effective SCM management can solely improve dairy yields and sustain milk production. One promising approach to SCM management is microbiome analysis, which provides insight into bacterial populations in dairy animals (Panchal et al. 2024; Zhu et al. 2024). Microbiome analysis provides a more comprehensive understanding of SCM and its microbial diversity than conventional techniques (Emon et al. 2024). Recent advancements in microbiome research have demonstrated that mastitis is not driven solely by individual pathogens, but by nuanced microbial interactions within the mammary gland (Panigrahi et al. 2025). Udder health depends on the milk microbiome, including commensal and pathogenic bacteria, while mastitis has been associated with dysbiosis, or microbial imbalance (Guo et al. 2024). Conventional culture‐dependent techniques often lack coverage of the full microbial diversity, whereas high‐throughput sequencing techniques, including 16S rRNA gene sequencing and metagenomics, provide comprehensive insight into the milk microbiota (Oikonomou et al. 2014). Gaining insight into these microbial communities can aid early disease detection, the development of probiotics, and the formulation of targeted therapeutic approaches to minimize reliance on antibiotics (Parente et al. 2020).

This study used 16S rRNA amplicon sequencing to identify bacteriome diversity in milk samples from cattle and buffalo across four different sample categories: M_C, M_B, C_H and B_H (Yasmin et al. 2025). Six pooled milk samples were analysed, yielding 423 clustered nucleotide sequences that provide significant insights into the microbial composition of milk in both infected and healthy states. Oxford Nanopore sequencing has higher raw error rates, and amplicon sequence variant (ASV)‐based denoising methods, such as DADA2, which require nearly error‐free reads, and were therefore not used (Callahan et al. 2016). Comparative studies have demonstrated that, despite its higher raw sequencing error rate, Oxford Nanopore technology yields microbiome profiles broadly comparable to those generated by short‐read sequencing platforms in 16S rRNA‐based analyses (Veselovsky et al. 2025). Therefore, taxonomic classification was performed using the EPI2ME workflow (Oxford Nanopore Technologies), which uses the k‐mer–based Kraken2 algorithm to assign taxonomy directly to individual reads by matching them to a reference database, rather than clustering into operational taxonomic units (OTUs) or ASVs.

Milk samples from healthy and mastitic cattle and buffalo exhibited significant microbial diversity. The diversity of these bacterial taxa across the four sample groups (M_C, M_B, C_H and B_H) offers preliminary insight into how host species and mastitis may influence the milk microbiome. Among the 120 identified bacterial families, about 15.0% were shared across all groups. However, there were no families solely associated with the M_C or M_B groups, suggesting that while mastitis may affect microbial composition at various taxonomic levels, bacterial families are generally distributed among different hosts and health conditions (Yasmin et al. 2025). At the genus level, 5.88% of the 272 identified genera were common to all groups, with the M_C group showing a higher number of unique genera (178) compared to the M_B group (71), suggesting potential but preliminary differences in microbial composition. In the dataset, the M_C group exhibited a greater number of unique genera than the M_B group, indicating a more pronounced observed compositional shift in cattle (Yasmin et al. 2025). However, this observation warrants cautious interpretation, as it reflects a descriptive trend derived from a pooled and limited pilot dataset rather than a definitive comparative conclusion. The microbial composition of milk from healthy and mastitic cattle and buffalo varied in taxonomic profiles. These differences may relate to host species and health status, potentially affecting the milk bacteriome (Yasmin et al. 2025). In the M_C samples, the predominant phyla were Firmicutes and Proteobacteria. Research from Indonesia, South Africa and Pakistan indicated that Firmicutes and Proteobacteria predominated in mastitic milk samples, reinforcing the consistency of these findings across multiple studies (Kusumawati et al. 2021; Khasapane et al. 2023; Salman et al. 2023b). Furthermore, the most common phylum in the M_B samples was Firmicutes. These findings on the bacterial composition of buffalo milk in SCM were previously reported by Catozzi et al. (2017) and Salman et al. (2023a). This suggests that the microbial communities in buffalo mastitis may differ slightly from those in cattle, likely due to factors unique to each species. Firmicutes, on the other hand, were also more prevalent in healthy milk samples (C_H: 79.48% and B_H: 97.31). This finding is consistent with previous research, including that of Catozzi et al. (2017) and Salman et al. (2023a). Therefore, Firmicutes may significantly influence the microbial population in both healthy and mastitic milk, with their relative abundance much higher in healthy milk.

In addition, the microbial communities in mastitic and healthy milk samples showed significant differences at the genus level. In the M_C samples, *Streptococcus* (55.36%) was the predominant genus, while *Lactococcus* (67.82%) was predominant in the M_B samples. Previous studies on cattle with SCM have reported different trends in bacterial prevalence compared with the current study. For example, African studies identified *Pseudomonas* as the most prevalent genus (Khasapane et al. 2023), while research in Pakistan found *Staphylococcus* to be dominant (Salman et al. 2023b). In a similar vein, earlier studies on mastitic buffaloes revealed disparities, with *Bacillus* identified as the most commonly occurring genus (Salman et al. 2023a). The genus composition in healthy milk samples exhibited unique patterns. In C_H milk, *Staphylococcus* (70.86%) appeared as the predominant genus, and the B_H milk was largely characterised by *Lactococcus* (83.94%). In comparison with other studies, the genus identified in healthy cattle milk in the present study was consistent with findings reported in Pakistan (Salman et al. 2023b). However, in healthy buffalo milk, the predominant genus observed in this study differed markedly from previous reports, in which *Acinetobacter* was identified as the dominant genus (Salman et al. 2023a). Differences in genus‐level composition between mastitic and healthy milk samples indicate that mastitis may involve changes in the milk bacteriome, including increased abundance of bacterial taxa potentially linked to disease progression. The relatively higher prevalence of *Streptococcus* in M_C samples aligns with previous reports that identify members of this genus as associated with mastitis pathophysiology. In contrast to the M_B samples, previous studies have reported varying predominant genera in milk samples affected by SCM, with *Corynebacterium* (11.67%) as the predominant genus in milk from cattle with mastitis (Kusumawati et al. 2021). Similarly, *Porphyromonas* was the predominant genus in the milk of buffalo with SCM, emphasizing the distinct species‐specific variations in the microbial communities associated with this condition (Catozzi et al. 2017). Variations like these could be shaped by elements such as sampling differences, experimental design and environmental conditions. Functional classification of the predicted bacterial communities indicated differences in the relative representation of functional groups among the sample groups. In the M_C group, dehalogenizer‐associated taxa were more prevalent (50.56%), whereas the M_B group showed a more even distribution of ammonia oxidizers (19.96%), sulphide oxidisers (19.93%) and dehalogenizers (19.88%). The control groups also exhibited distinct community compositions, with dehalogenizer‐associated taxa constituting a considerable proportion of the bacterial community (C_H: 25.67%, B_H: 21.46%). Differences in microbial dominance suggest that mastitis may cause modest shifts in the milk microbiome of cattle and buffalo, possibly reflecting physiological or immunological differences between the species. These findings provide preliminary predictive insights into the milk microbiome. Further validation using deeper sequencing and targeted in vitro and in vivo studies is required to confirm the functional roles and biological relevance of the observed microbial patterns.

## 5 Conclusion

This study offers preliminary insights into the microbial composition of milk from cattle and buffalo, with a specific focus on the potential effects of SCM. Through 16S rRNA amplicon sequencing, distinct differences in bacteriome composition were identified between mastitic and healthy milk, particularly at the genus level. At the genus level, *Streptococcus* was more prevalent in mastitic cattle milk, while *Lactococcus* and *Acidipropionibacterium* were more abundant in buffalo milk. These results provide an initial overview of microbial patterns in milk and underscore the need for further comprehensive sequencing and functional studies to clarify the role of specific microbial communities in mastitis. Although this pilot study suggests a potential association between SCM and microbial shifts, these findings remain preliminary due to the limited sample size and pooled sampling design. Accordingly, microbiome profiling cannot yet be considered suitable for diagnostic use. Vigorous evaluation of its diagnostic potential will require larger, well‐powered studies with individual‐level sampling and appropriate statistical validation. Additional validation through targeted in vitro and in vivo studies is necessary to confirm the functional roles and biological significance of the observed microbial patterns.

### 5.1 Study Limitations

The analysis was based on pooled milk samples, which may obscure individual‐level variation in microbial composition. The limited sample size and exclusive focus on taxonomic profiling may have constrained the detection of finer‐scale microbial patterns. Future research should incorporate larger cohorts, longitudinal sampling, and functional metagenomic analyses to clarify the ecological and biological significance of the observed microbial communities. Another limitation of this study is that the DNA extraction kit used was not specifically validated for milk samples. Although the same extraction protocol was applied consistently across all samples, potential extraction‐efficiency bias may have influenced the relative recovery of some bacterial taxa.

## Author Contributions

**Md. Habib Ullah Masum**: methodology, software, data curation, investigation, validation, formal analysis, visualization, writing – original draft, writing – review and editing. **Maksudur Rahman Nayem**: software, data curation, validation, visualization. **Ahmad Abdullah Mahdeen**: methodology, data curation, formal analysis. **Sumaia Sultana**: methodology, data curation, formal analysis. **Abanti Barua**: conceptualization, software, investigation, validation, supervision, writing – review and editing.

## Funding

The authors have nothing to report.

## Ethics Statement

The Ethics Committee of Noakhali Science and Technology University (NSTUEC) conducted a thorough assessment and granted approval for the experimental protocols related to this study (reference number: NSTU/SCI/EC/2023/126A). The herders and animal owners present at the local dip location gave their verbal consent to participate in the study. Before the sampling process, the animals rested and settled down. All samples were taken before commencing any antibiotic treatment.

## Conflicts of Interest

The authors declare no conflicts of interest.

## Supporting information




**Supporting File 1**: vms371154‐sup‐0001‐DataS1.xlsx

**Supporting File 2**: vms371154‐sup‐0002‐DataS2.xlsx

**Supporting File 3**: vms371154‐sup‐0003‐TableS1.docx

**Supporting File 4**: vms371154‐sup‐0004‐TableS2.docx

**Supporting File 5**: vms371154‐sup‐0005‐FigureS1.docx

## Data Availability

Sequence data that support the findings of this study have been deposited in the National Center for Biotechnology Information (NCBI) Sequence Read Archive (SRA) with the accession PRJNA1113616.
