# Supplementary material for: Characterizing the Milk Microbiome in Subclinical Mastitis: A Pilot 16S rRNA‐Based Study in Cattle and Water Buffalo
Source: Vet Med Sci. 2026 Aug 5;12(5):e71154. doi: 10.1002/vms3.71154 (PMC13439521; doi:10.1002/vms3.71154)
Supplement: Supplementary file 1 [file VMS3-12-e71154-s003.docx]

**Supplementary Fig. S1.** Rarefaction curves generated after raw sequence filtering and normalization of the dataset.
