# Supplementary material for: Characterizing the Milk Microbiome in Subclinical Mastitis: A Pilot 16S rRNA‐Based Study in Cattle and Water Buffalo
Source: Vet Med Sci. 2026 Aug 5;12(5):e71154. doi: 10.1002/vms3.71154 (PMC13439521; doi:10.1002/vms3.71154)
Supplement: Supplementary file 2 [file VMS3-12-e71154-s004.docx]

| Taxa | Sample group (pooled samples) | | | |
| --- | --- | --- | --- | --- |
|  | M_C | M_B | C_H | B_H |
| Phylum (n = 11) | 11 | 7 | 7 | 6 |
| Class (n = 26) | 18 | 15 | 9 | 9 |
| Order (n = 58) | 34 | 33 | 15 | 18 |
| Family (n = 120) | 60 | 55 | 30 | 25 |
| Genus (n = 272) | 103 | 97 | 37 | 27 |

**Supplementary Table S2**: Taxonomic information of microbiomes in the four sample groups (M_C, M_B, C_H and B_H).
