# Supplementary material for: Characterizing the Milk Microbiome in Subclinical Mastitis: A Pilot 16S rRNA‐Based Study in Cattle and Water Buffalo
Source: Vet Med Sci. 2026 Aug 5;12(5):e71154. doi: 10.1002/vms3.71154 (PMC13439521; doi:10.1002/vms3.71154)
Supplement: Supplementary file 3 [file VMS3-12-e71154-s005.docx]

**Supplementary Table S1:** Sample information and SRA accession numbers for 16S rRNA amplicon sequences are provided for bacterial taxa and the four sample groups (M_C, M_B, C_H, and B_H).

| Sample group | Pooled Sample ID | Collection site | Coordinate | Category | SRA accessions |
| --- | --- | --- | --- | --- | --- |
| M_C | M1 | Noakhali, Bangladesh | 22.8724 N 91.0973 E | Mastitis Cattle | SRR29085943 |
|  | M2 | Noakhali, Bangladesh | 22.8724 N 91.0973 E | Mastitis Cattle | SRR29085942 |
| M_B | M3 | Noakhali, Bangladesh | 22.8724 N 91.0973 E | Mastitis Buffalo | SRR29085941 |
|  | M4 | Noakhali, Bangladesh | 22.8724 N 91.0973 E | Mastitis Buffalo | SRR29085940 |
| C-H | C-H | Noakhali, Bangladesh | 22.8724 N 91.0973 E | Mastitis Cattle | SRR29085939 |
| B-H | B-H | Noakhali, Bangladesh | 22.8724 N 91.0973 E | Mastitis Buffalo | SRR29085938 |
